# The current state of cannabinoids in orofacial pain

**DOI:** 10.1016/j.clinsp.2024.100528

**Published:** 2024-10-22

**Authors:** Eduardo Grossmann, Sigmar de Mello Rode, Claudia Herrera Tambeli, João Paulo Colesanti Tanganeli, Denise Sabbagh Haddad

**Affiliations:** a Universidade Federal do Rio Grande do Sul, Porto Alegre, RS, Brazil; b São José dos Campos Dentistry Course, Universidade Estadual Paulista (UNESP), São José dos Campos, SP, Brazil; c Pain Studies Laboratory, Universidade Estadual de Campinas, Campinas, SP, Brazil; d Associação dos Cirurgiões Dentistas do Estado de São Paulo, Faculdade de Odontologia, São Paulo, SP, Brazil

Orofacial pain, which includes a range of conditions affecting the face and mouth, is a complex and often debilitating condition and has long been a challenge in the medical community due to its multifaceted nature. With advancements in cannabinoid research, particularly in relation to the endocannabinoid system, there is increasing interest in how cannabinoids can be leveraged for pain management. While traditional pharmacological approaches such as opioids and NSAIDs have long been utilized, their limitations have prompted the exploration of alternative therapies, including cannabinoids. Recent studies suggest that cannabinoids may offer new therapeutic avenues for managing orofacial pain by targeting specific receptors and modulating pain pathways.

The endocannabinoid system, composed of Cannabinoid receptors (CB1 and CB2), endogenous ligands, and enzymes responsible for the synthesis and degradation of endocannabinoids, plays a crucial role in pain modulation and neuroinflammation.^1^^,^^2^ Cannabinoid receptor 1 (CB1) is primarily located in the Central Nervous System (CNS), while CB2 receptors are predominantly found in peripheral tissues and immune cells. Research has shown that activating these receptors can influence pain signaling pathways, making them attractive therapeutic targets for managing chronic pain, including orofacial conditions.^3^

A significant focus of cannabinoid research in recent years has been on their potential to alleviate neuropathic pain, a common feature of many orofacial disorders. Studies have shown that cannabinoids, particularly THC and CBD, exert analgesic effects by modulating pain perception both centrally and peripherally.^4^ For instance, transdermal applications of CBD have demonstrated a muscle-relaxing effect in patients suffering from Temporomandibular Disorders (TMD), a form of orofacial pain.^3^ In addition to their direct action on CB1 and CB2 receptors, cannabinoids have been found to influence other pathways involved in pain transmission. The orphan receptor GPR55, which has been identified as a novel cannabinoid receptor, plays a role in modulating pain responses.^5^

This broad spectrum of activity suggests that cannabinoids might offer therapeutic benefits beyond traditional painkillers, with fewer side effects, particularly when it comes to chronic pain management. The use of cannabinoids in treating orofacial pain is not without controversy. While some studies suggest promising results, such as a reduction in pain severity and improvement in quality of life, others caution about the variability in patient response and the potential for adverse effects, especially with THC-dominant products.^6^

A systematic review on the use of cannabis-based medicines for orofacial pain management highlights the need for more robust, randomized controlled trials to establish clear clinical guidelines.^7^ Recent studies have also explored the therapeutic potential of cannabinoids in specific orofacial pain conditions like trigeminal neuralgia and Burning Mouth Syndrome (BMS). Trigeminal neuralgia, a severe form of facial pain, has been reported to respond to cannabis-based treatments such as nabiximols, which combine THC and CBD in a spray formulation.^8^ Meanwhile, BMS, a chronic pain condition characterized by a burning sensation in the mouth, has shown a reduction in symptoms with cannabis sativa oil, further underscoring the potential for cannabinoids in niche pain conditions.^9^

Despite these promising developments, there are several barriers to the widespread use of cannabinoids in orofacial pain treatment. The legal and regulatory environment surrounding cannabis and cannabis-based products varies widely across countries and even regions within countries. Furthermore, the psychoactive effects of THC, particularly at higher doses, limit its acceptability for long-term use in certain patient populations.^10^

Looking forward, ongoing research is essential to clarify the role of cannabinoids in orofacial pain. Newer compounds, such as synthetic cannabinoids and selective CB2 agonists, may offer a way to harness the analgesic properties of cannabinoids without the psychoactive effects associated with THC.^11^ Additionally, advancements in the understanding of the endocannabinoid system's broader interaction with pain pathways may pave the way for more targeted therapies.^12^

The exploration of cannabinoids as a therapeutic option for orofacial pain is a rapidly evolving field. The ECS, with its widespread influence on pain and inflammation, offers a promising target for cannabinoid-based interventions. Current research suggests that cannabinoids, particularly when used in combination with other treatments, may provide relief for patients suffering from chronic orofacial pain. However, further studies are necessary to fully understand the mechanisms, efficacy, and safety of these compounds. As the medical community continues to investigate the complexities of the ECS and cannabinoids, there is hope that these therapies may offer a new paradigm in orofacial pain management.

## Declaration of competing interest

The authors declare no conflicts of interest.
